# Detection of *Trypanosoma* spp. and *Leishmania* spp. in bats from the department of Atlántico, Colombia

**DOI:** 10.17843/rpmesp.2026.431.15494

**Published:** 2026-03-27

**Authors:** Brendy Alcira Padilla-Obregón, Iván Benavides-Céspedes, Yeisson Cera-Vallejo, Daisy Lozano-Arias, Carlos Mario Meléndez, Roberto Garcia-Alzate

**Affiliations:** 1 Biology Program, Faculty of Basic Sciences, Universidad del Atlántico, Puerto Colombia, Colombia.; 2 Department of Animal Science, Faculty of Veterinary Sciences, Universidad de Concepción, Chillán, Chile.; 3 Basic and Clinical Research Group in Health Sciences, Faculty of Health Sciences, Fundación Universitaria San Martin, Puerto Colombia, Colombia.; 4 Chemistry Program, Faculty of Basic Sciences, Universidad del Atlántico, Puerto Colombia, Colombia.

**Keywords:** Bats, Host, Natural Infection, Trypanosoma, Leishmania

## Abstract

**Objective.:**

To determine the frequency of *Trypanosoma* spp. and *Leishmania* spp. infection in bats captured in the municipalities of Piojó, Tubará, and Usiacurí, in the department of Atlántico, in the northern Colombian Caribbean region.

**Materials and methods.:**

Between August 2022 and April 2023, bats were captured using mist nets; these were taxonomically identified, and their sex, reproductive stage, and relative age were established. Blood samples were obtained from the brachial vein. The identification of *Trypanosoma* spp. was performed through parasitological and molecular diagnosis; DNA was extracted, and the kinetoplast region (kDNA) was amplified. For the identification of *Leishmania* spp., the β-tubulin region was amplified.

**Results.:**

From 110 bats analyzed, an overall infection frequency of 14.50% (16/110) was determined. The presence of *Trypanosoma* spp. was detected in 14.50% (16/110) of the individuals and *Leishmania* spp. in 10.00% (11/110). Coinfection was identified in 11 specimens, equivalent to a frequency of 10.00% of the total sample. The coinfected bats belonged to the species *Artibeus lituratus*, *Artibeus jamaicensis*, and *Phyllostomus hastatus*.

**Conclusions.:**

This study constitutes the first report on the frequency of trypanosomatid coinfection in bats from the department of Atlántico.

## INTRODUCTION

Bats belong to the Order Chiroptera, considered the second most diverse order of mammals, with 1474 recognized species (https://www.mammaldiversity.org). This diversity is reflected in their ecological functions (pollination, seed dispersal, and arthropod control) derived from their feeding preferences ^1^. These characteristics, along with flight, longevity, and social behavior, make bats ideal hosts for viruses, bacteria, and protozoa, highlighting the hemoflagellates of the family Trypanosomatidae, which includes genera of medical and veterinary importance (genera *Trypanosoma* and *Leishmania*) as etiological agents of diseases in humans and other animals ^2^^-^^4^.

Within the genus *Trypanosoma*, the species *Trypanosoma* (*Schizotrypanum*) *cruzi* (Kinetoplastea, Trypanosomatidae) stands out, presenting seven discrete typing units (DTUs) TcI - TcVI, including a bat-specific DTU called TcBat, which differ in their geographic distribution and transmission cycles ^5^. Furthermore, *T. cruzi* can complete part of its life cycle in mammals from up to nine orders, including Chiroptera ^6^. It is also the etiological agent of American trypanosomiasis (AT) or Chagas disease (CD), considered one of the main causes of morbidity, disability, and mortality, primarily in the Americas, where it is endemic in 21 countries according to the World Health Organization (WHO) (2025) ^7^.

The genus *Leishmania* includes 21 species that affect humans and other vertebrates, causing cutaneous leishmaniasis (CL), mucocutaneous leishmaniasis (MCL), and visceral leishmaniasis (VL) ^4^. In Colombia, Marinkelle ^8^ was the first to point out the importance of bats as hosts for *T. cruzi*, reporting 233 positive hemocultures ^8^. More recent studies, such as those by Carrasquilla et al. ^4^, Benavides-Céspedes et al. ^9^, and Matiz-González et al. ^10^, provide updated data on the role of bats as hosts for trypanosomatids.

Trypanosomatid infection in bats derives from trophic and biological interaction with their vectors. While *Trypanosoma* spp. is transmitted by the ingestion of infected triatomines or contact with their feces, *Leishmania* spp. is transmitted through the bite of sand flies during blood-feeding, consolidating a multidirectional dynamic in ecosystems ^4^^,^^6^^,^^8^^-^^10^. In Colombia, for epidemiological week 53 of 2025, 12 cases of acute CD (one in Atlántico) and 5721 cases of leishmaniasis were reported ^11^.

In the department of Atlántico, vectors for *Trypanosoma* spp. such as *Panstrongylus geniculatus* and for *Leishmania* such as *Lutzomyia longipalpis*, *Lu. evansi*, and *Lu. gomezi* have been identified in several municipalities, considered as risk factors ^12^^,^^13^; the presence of *Trypanosoma* spp. has also been detected in bats of the species *Molossus molossus* and *Noctilio albiventris*^9^. Given the increasing human contact with wild animals, the objective of this study was to determine the frequency of infection by *Trypanosoma* spp. and *Leishmania* spp. in bats from peridomestic ecotopes in the department of Atlántico.

**KEY MESSAGES**

Motivation for the study. In the department of Atlántico, the first report of trypanosomatids in bats was documented; therefore, it is necessary to expand this knowledge to other municipalities in the region.

Main findings. This study constitutes the first report of the frequency of trypanosomatid coinfection and *Leishmania* spp. in bats of the genera *Artibeus* and *Phyllostomus* in the department of Atlántico.

Implications.The identification of bats infected with *Trypanosoma* spp. and *Leishmania* spp. in municipalities with the presence of biological vectors for these parasites represents a potential risk to public health, due to their possible role in zoonotic transmission.

## MATERIALS AND METHODS

### Study Type and Area

The present study is observational, descriptive, and cross-sectional; sampling was conducted between August 2022 and April 2023 in peridomestic ecotopes of three municipalities in the department of Atlántico (10° 39’ 00’’ N, 74° 58’ 00’’ W), located in northern Colombia, at 123 m a.s.l. with an average temperature of 27.1 °C and precipitation of 1396 mm ^14^. The municipalities were Usiacurí, Piojó, and Tubará; these were chosen for presenting risk factors such as the presence of host mammals (unpublished data) and triatomines positive for *T. cruzi*^12^.

The study area is under the Köppen-Geiger climate classification as equatorial savanna with dry winter (Aw); under this framework, the municipality of Piojó (10º 45’ 01” N, 75º 06’ 27” W) presents an average annual temperature of 26 °C and an altitude of 240 m a.s.l., with an average annual precipitation of 1200 mm and a bimodal regime composed of two rainy periods (May-June and August-November) and two dry periods (December-April and June-July), making it one of the rainiest and highest municipalities in Atlántico. Usiacurí (10° 44’ 46’’ N, 74° 59’ 02’’ W), located at an altitude of 95 m a.s.l., records an average annual temperature between 25 °C and 30 °C and precipitation of 980 mm annually, with rainfall peaks in the periods of May-June and September-October.

**Figure 1:**
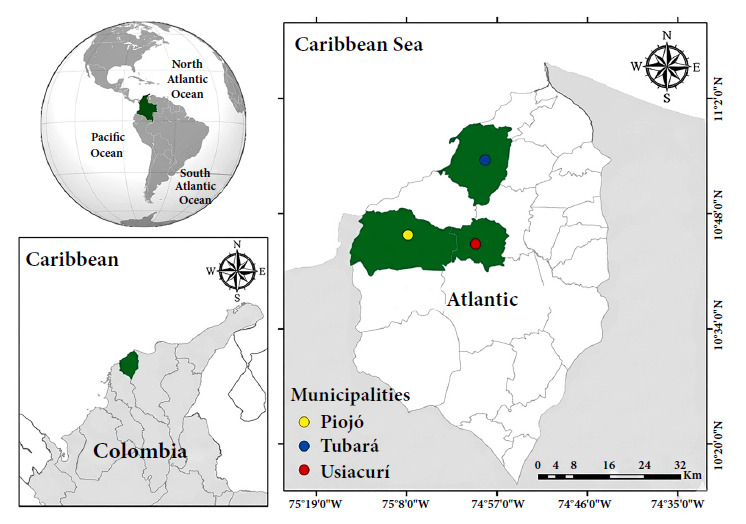
Geographic distribution of the three municipalities where bat species were captured in the Department of Atlántico.

Finally, Tubará (10° 52’ 27’’ N, 74° 58’ 43’’ W), located at 192 m a.s.l., records an average annual temperature between 26 °C and 29 °C and precipitation ranging between 379 mm and 1025 mm annually; its rainy season begins in April and ends in early December, with September, October, and November being the months of highest rainfall (Figure 1).

### Capture, Taxonomic Identification, Blood Sampling, and Marking of Bats

Two visits of two consecutive nights per municipality were conducted, totaling 12 nights. For the capture of bats, two mist nets of 12 x 2.5 m with a 3 × 3 cm mesh (BioWed, ®) were used, which were extended between 18:00 and 23:00 hours at two specific points (selected through pilot visits) of the peridomestic ecotope for each municipality, with a distance between nets of 25 meters; each point was geolocated with a GPS (Garmin Etrex 20). These were monitored every 15 minutes to minimize potential stress on the animals ^15^. Individuals extracted from the nets were placed individually in 20 × 20 × 20 cm cotton bags for subsequent identification using the taxonomic key of Díaz *et al*. ^16^. Sex, relative age, and reproductive status were determined according to the indications of Kunz and Wemmer ^17^.

For detection, 100 μL of blood was collected from the brachial vein, divided into two 50 μL aliquots: one in EDTA at 4 °C for molecular analysis and another for in situ parasitological examination. The latter consisted of searching for hemoflagellates compatible with *Trypanosoma* spp. ^18^ using thick film (Giemsa) and bright-field microscopy (40X). Following sampling, micro-perforations were made in the right patagium to identify individuals and avoid recaptures before their release.

Once it was verified that the bat was in adequate condition to fly, it was released near the capture site. Pregnant and lactating females were excluded from the parasitological and molecular analyses. Data for each individual were recorded in a proprietary sheet. This research was approved by the Ethics Committee of the Universidad del Atlántico under code 02-III-2021, in accordance with resolution 1214 of the National Authority for Environmental Licenses (ANLA) for non-commercial scientific research in Puerto Colombia.

### Parasitological and Molecular Detection

DNA extraction from blood samples stored in EDTA tubes was performed with the HMW Wizard® purification kit (Promega Corporation: Madison, WI, USA), following the manufacturer's instructions starting from 50 μL of blood and eluted in a final volume of 20 μL. Genomic DNA concentration was determined using the EPOCH 2NS spectrophotometer (BioTeck Instruments, Inc: USA). Molecular detection of *Trypanosoma* spp. was performed through PCR amplification of the conserved region of the kinetoplast minicircle using primers 121 (AAA-TAA-TGT-ACG-GG(T/G)-GAG-ATG-CAT-GA) and 122 (GGT-TCG-ATT-GGG-GTT-GGT-GTA-ATA-TA), which amplify a 330 bp band ^19^. For molecular detection of the genus *Leishmania*, a PCR targeting the β-tubulin region was performed using primers TUB 1 (ATG-CGT-GAG-ATC-GTT-TCC) and TUB 6 (GGC-GGC-CTG-CAT-CAT); samples were considered positive when a 900 bp band was amplified ^20^. Ultrapure water was used as a No Template Control (NTC) in each PCR reaction. Genomic DNA from a local reference strain of *T. cruzi* and the WHO reference strain *L. (Viannia) panamensis* (MHOM/PA/71/LS94) were used as positive controls for the detection of *Trypanosoma* spp. and *Leishmania* spp., respectively. Controls for each molecular marker were previously performed to ensure no cross-reactions existed.

PCR reactions were standardized using the PCR Master Mix (Promega®). For the detection of *Trypanosoma* spp., a final volume of 15 μL was used (5 μL of template DNA), adding a final concentration of 0.016 U/μL of Taq DNA polymerase, 0.66 μM of each primer, and a supplementation of 1.6 mM of MgCl₂ in addition to the 1.5 mM present in the commercial mix. For the genus *Leishmania*, the final volume was 20 μL (5 μL of template DNA), adjusting the concentrations to 0.015 U/μL of Taq DNA polymerase, 1.0 μM of each primer, and a supplementation of 1.25 mM of MgCl₂. In both cases, nuclease-free water (Promega®) was used to complete the final volume.

Each PCR was performed in a TC-9639 thermal cycler (Benchmark SCIENTIFIC, Sayreville, NJ, USA) under the following conditions: For *Trypanosoma* spp. initial denaturation at 94 ºC for 10 minutes, 35 cycles of denaturation at 94 ºC/1 minute, hybridization at 63 ºC/1 minute, extension at 72 ºC/1 minute, and final extension at 72 ºC for 10 minutes ^19^. For the genus *Leishmania*: initial denaturation at 94 °C for 5 minutes, 40 cycles of denaturation at 94 °C/30 seconds, hybridization at 62 °C/ 30 seconds, extension at 72 °C/30 seconds, and a final extension at 72 °C/10 minutes ^20^.

The products obtained were visualized by horizontal electrophoresis (80V/60 minutes) on a 2.0% agarose gel, stained with ethidium bromide diluted in 1X TAE buffer for 20 minutes for visualization and photodocumentation in the iBright™ FL1500 system (Thermo Fisher Scientific Inc: MA, USA); a molecular marker of 100 to 1,000 bp (Biotech) was used as a reference.

### Data Analysis

Total sampling effort/per night and capture success were calculated according to general recommendations for mist net sampling; infection frequency was calculated in percentages using the standard formula, while alpha diversity was estimated using Hill's series of abundance and diversity orders (with q=0 richness is obtained directly, for q=1 abundant species, and for q=2 very abundant or dominant species) ^21^. Rarefaction curves were performed for each of the orders, which were interpolated and extrapolated to the minimum and maximum number of individuals collected in each locality, allowing comparison of sites with different numbers of individuals, and whose shading indicates 95% confidence intervals ^22^. All alpha diversity calculations were performed in Excel and the iNEXT package of the R statistical program ^23^.

Fisher's exact test (two-tailed) was used to evaluate whether the total trypanosomatid infection frequencies between the two zones (Usiacurí and Tubará) were significantly different. Analyses were performed in IBM SPSS Statistics version 25, and a 95% confidence level (α =0.05) was used. Infection proportions by area, the difference in proportions, and its 95% confidence interval were reported.

### Ethical Aspects

All applicable international, national, and/or institutional guidelines for the care and use of animals were followed. The study was approved by the Ethics Committee of the Universidad del Atlántico, Puerto Colombia, Colombia, with code 02-IV-2022.

## RESULTS

### Total Sampling Effort, Sampling Effort per Night, Capture Success, and Bat Species Diversity

The total sampling effort of the study was 3600 h/net. Regarding the sampling effort per night, this was 150 h/net, being homogeneous for each zone, while the capture success for Usiacurí was 0.34 ind/h-net, in Tubará, it was 0.26 ind/h-net, and in the municipality of Piojó, it was 0.15 ind/h-net.

A total of 114 bats were captured (no recaptures were recorded) (N=114) represented in three families and 10 species. The family *Phyllostomidae* was the most abundant, representing 96.50% (110/114) of the total captures; the families *Vespertilionidae* (2.62% - 3/114) and *Mormoopidae* (0.88% - 1/114) were poorly represented. The most abundant species were *A. lituratus* (41.23% - 47/114) and *A. jamaicensis* (35.09% - 40/114), with the genus *Artibeus* contributing 76.32% of the total individuals; the distribution of bats by municipality can be observed in Table 1.

**Table 1:** Trophic guild, reproductive status, and age of the species collected in the study areas.

Area	Family	Species	Trophic Guild	Reproductive status									Age F				Age M				Total
				SAM	NSAM	Total	SAF	NSAF	PF	LF	FWO	Total	A	SA	J	Total	A	SA	J	Total	
Piojó	*Phyllostomidae*	*Artibeus lituratus*	P.F	7	1	8	2	0	1	0	0	2	7	0	1	8	1	1	0	2	10
		*Glossophaga soricina*	Nc	5	0	5	2	2	0	1	0	4	5	0	0	5	1	1	2	4	9
		*Desmodus rotundus*	Hm	2	0	2	0	0	0	0	0	0	2	0	0	2	0	0	0	0	2
	*Vespertilionidae*	*Myotis riparius*	In	0	0	0	2	0	0	0	0	2	0	0	0	0	2	0	0	2	2
	Total			14	1	15	6	2	1	1	0	8	14	0	1	15	4	2	2	8	23
Tubará	*Phyllostomidae*	*Artibeus lituratus*	P.F	6	0	6	10	2	0	0	0	12	5	1	0	6	8	2	2	12	18
		*Artibeus jamaicensis*	P.F	5	3	8	3	3	0	0	0	6	5	0	3	8	3	0	3	6	14
		*Carollia perspicillata*	Fr	1	0	1	0	4	0	0	0	4	1	0	0	1	0	0	4	4	5
		*Glossophaga soricina*	Nc	0	0	0	0	1	0	0	0	1	0	0	0	0	0	0	1	1	1
	*Vespertilionidae*	*Myotis riparius*	In	0	0	0	0	1	0	0	0	1	0	0	0	0	0	0	1	1	1
	*Mormoopidae*	*Pteronotus gymnonotus*	In	0	1	1	0	0	0	0	0	0	0	0	1	1	0	0	0	0	1
	Total	* *		12	4	16	13	11	0	0	0	24	11	1	4	16	11	2	11	24	40
Usiacurí	*Phyllostomidae*	*Artibeus jamaicensis*	P.F	9	4	13	7	6	0	0	0	13	7	2	4	13	5	2	6	13	26
		*Artibeus lituratus*	P.F	9	2	11	4	4	0	1	0	8	8	1	2	11	4	0	4	8	19
		*Phyllostomus hastatus*	Om	1	0	1	0	0	0	0	0	0	1	0	0	1	0	0	0	0	1
		*Phyllostomus discolor*	In	1	0	1	0	0	0	0	0	0	1	0	0	1	0	0	0	0	1
		*Glossophaga soricina*	Nc	0	0	0	2	1	1	0	0	3	0	0	0	0	1	1	1	3	3
		*Uroderma convexum*	Fr	1	0	1	0	0	0	0	0	0	1	0	0	1	0	0	0	0	1
	Total			21	6	27	13	11	1	1	0	24	18	3	6	27	10	3	11	24	51

M: male, F: female, SAM: sexually active male, NSAM: non-sexually active male, SAF: sexually active female, NSAF: non-sexually active female, PF: pregnant female, LF: lactating female, FWO: female with offspring, A: adult, SA: subadult, J: juvenile. P.F: predominantly frugivorous, Nc: nectarivorous, Hm: hematophagous, In: insectivorous, Fr: frugivorous, Om: omnivorous.

The majority of captured bats were adults (59.60%, 68/114), followed by juveniles (30.70%, 35/114) and subadults (9.60%, 11/114). Regarding sex, 50.80% (58/114) were males and 49.10% (56/114) were females. No females with offspring were found, but two lactating and two pregnant females were captured. Details by area are presented in Table 1.

**Figure 2:**
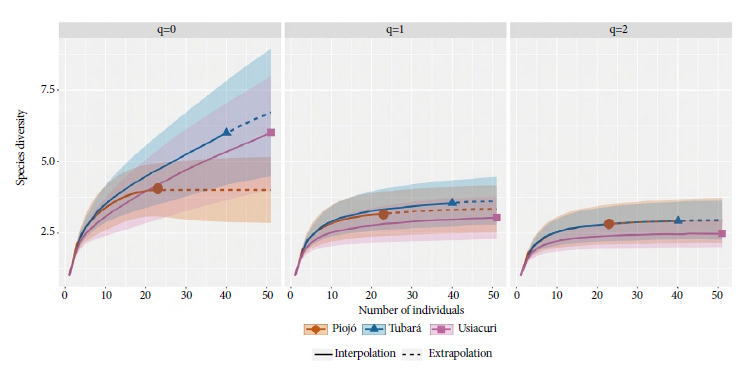
Rarefaction curves for each diversity order in the municipalities of Piojó, Tubará, and Usiacurí. The solid line represents the interpolated or observed region, the dotted line represents the extrapolated region, and the shading around each curve corresponds to the 95% confidence interval (CI).

Rarefaction curves showed that richness (q=0) for Usiacurí and Tubará was homogeneous, with six species, and with greater sampling effort more could be recorded. Piojó presented the lowest richness, with four species, and its curve stabilized, suggesting that no more species would be added with further sampling. Differences in richness between municipalities were not statistically significant (α = 0,05). Regarding abundance (q=1), Tubará and Piojó showed similarities, without significant differences; the number of abundant species was adequate in all municipalities. For the effective number of dominant species (q=2), Piojó and Tubará also showed similarities, with greater evenness compared to Usiacurí, although without significant differences (Figure 2).

### Infection Frequency

Of the 114 captured bats, 110 (96.50%) were analyzed due to the exclusion criteria for pregnant and lactating females mentioned above. Thus, using the parasitological method, the infection frequency obtained was 7.30% (8/110), distributed between the municipalities of Usiacurí (62.50% - 5/8) and Tubará (37.50% - 3/8); individuals with the presence of blood trypomastigotes belonged to the species *P. hastatus*, *A. jamaicensis*, and *Pteronotus gymnonotus*; details are specified in Table 2.

**Table 2:** Frequency of infection overall and by bat species obtained in each of the study areas.

Area	% Parasitological (n/N) 95% CI	% Molecular (n/N) 95% CI	Species	*Bloodborne Trypanosoma*	*Trypanosoma* (Mol)	*Leishmania*	Coinfection
Usiacurí	10.20 (5/49) 4.43‒21.97	22.45 (11/49) 13.01‒35.93	*A. jamaicensis*	15.38 (4/26) 6.14‒33.53	23.08 (6/26) 10.91‒42.26	23.08 (6/26) 10.91‒42.26	23.08 (6/26) 10.91‒42.26
			*A. lituratus*	0.00 (0/18) 0.00‒17.59	22.22 (4/18) 8.97‒45.18	22.22 (4/18) 8.97‒45.18	22.22 (4/18) 8.97‒45.18
			*P. hastatus*	100.00 (1/1) 20.65‒100.00	100.00 (1/1) 20.65‒100.00	100.00 (1/1) 20.65‒100.00	100.00 (1/1) 20.65‒100.00
Tubará	7.50 (3/40) 2.58‒19.85	12.50 (5/40) 5.45‒25.95	*A. jamaicensis*	14.29 (2/14) 4.00‒40.03	7.14 (1/14) 1.27‒31.48	0.00 (0/14) 0.00‒21.50	0.00 (0/14) 0.00‒21.50
			*A. lituratus*	0.00 (0/18) 0.00‒17.59	11.11 (2/18) 3.11‒32.80	0.00 (0/18) 0.00‒17.59	0.00 (0/18) 0.00‒17.59
			*C. perspicillata*	0.00 (0/5) 0.00‒45.93	20.00 (1/5) 3.62‒62.45	0.00 (0/5) 0.00‒45.93	0.00 (0/5) 0.00‒45.93
			*Pt. gymnonotus*	100.00 (1/1) 20.65‒100.00	100.00 (1/1) 20.65‒100.00	0.00 (0/1) 0.00‒79.35	0.00 (0/1) 0.00‒79.35
Piojó	0.00 (0/21) 0.00‒15.33	0.00 (0/21) 0.00‒15,33		0.00 (0/21) 0.00‒15.33	0.00 (0/21) 0.00‒15.33	0.00 (0/21) 0.00‒15.33	0.00 (0/21) 0.00‒15.33
Total	7.27 (8/110) 3.73‒13.71	14.55 (16/110) 9.13‒22.33		7.27 (8/110) 3.73‒13.71	14.55 (16/110) 9.13‒22.33	10.00 (11/110) 5.66‒17.04	10.00 (11/110) 5.66‒17.04

95% CI: 95% Confidence Interval calculated using the Wilson method.

**Figure 3:**
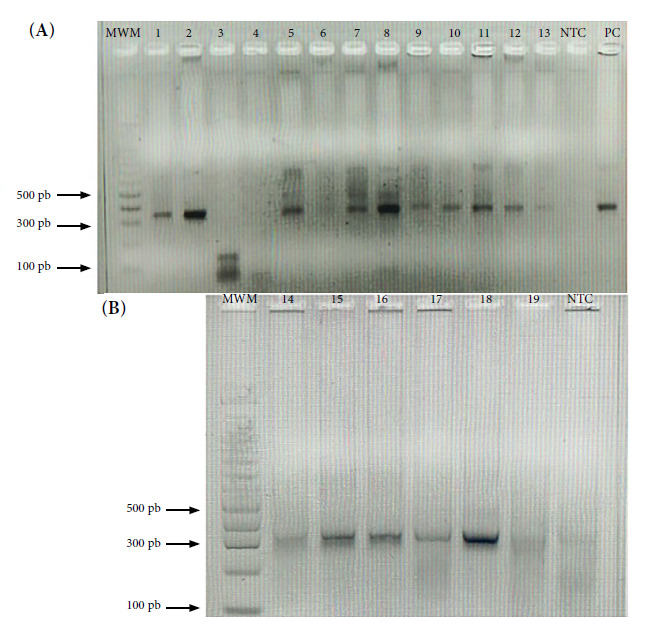
(A) 2% agarose gel in 1X TAE stained with ethidium bromide. PCR amplification of kDNA using 121-122. Positive samples: (1,2,5,7-13). (B) 2% agarose gel in 1X TAE stained with ethidium bromide. PCR amplification of kDNA using 121-122. Positive samples: (14-19). MWM: molecular weight marker. NTC: negative control (ultrapure water). PC: positive control (genomic DNA from a local reference strain of *Trypanosoma cruzi*-DVP50).

Regarding molecular detection, the frequency of infection by trypanosomatids was 14.50% (16/110). All of these positive individuals (100%) presented infection by *Trypanosoma* spp. (Figure 3A-B). Furthermore, in 11 of these 16 specimens, the presence of *Leishmania* spp. was detected (samples 1-11, Figure 4), representing a prevalence of 10.00% (11/110) for this genus. Notably, all positive cases for *Leishmania* spp. corresponded to coinfection events with *Trypanosoma* spp.

**Figure 4:**
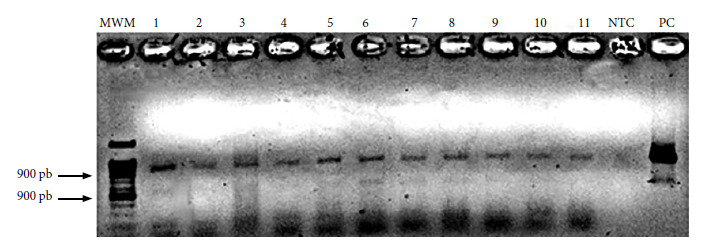
4. 2% agarose gel in 1X TAE stained with ethidium bromide. PCR amplification of TUB1/TUB6. Positive samples: 1-11. NTC: negative control (ultrapure water). PC: positive control (*Leishmania (Viannia) panamensis*- MHOM/PA/71/LS94).

### Comparison of Infection Frequencies between Study Areas

Regarding the frequency of infection and coinfection by zones, the municipality of Usiacurí presented the highest overall infection (trypanosomatids) and coinfection frequency with 22.45% (11/49), followed by the municipality of Tubará, where the overall infection frequency was 12.50% (5/40), with no cases of coinfection. Finally, the municipality of Piojó recorded no positive individuals. The species with the highest infection frequency were *P. hastatus* and *Pt. gymnonotus*; the species *P. hastatus* also presented the highest coinfection frequency; all details are grouped in Table 2.

Comparing the trypanosomatid infection frequency between bats from Usiacurí (22.40%; 11/49) and Tubará (12.50%; 5/40) using Fisher's exact test (two-tailed), no statistically significant difference was detected (p=0.332). The proportion was higher in Usiacurí; the difference in proportions was 0.0995 (95% CI: −0.0610 to 0.2600).

## DISCUSSION

In the present study, an overall trypanosomatid infection frequency of 14.55% was determined using molecular techniques, a figure that doubled the detection by direct microscopy (7.27%). The finding of 10.00% coinfection between *Trypanosoma* spp. and *Leishmania* spp. in generalist bats (*A. lituratus*, *A. jamaicensis*, and *P. hastatus*) stands out, constituting the first record of natural coinfection and the first detection of *Leishmania* spp. in bats for the department of Atlántico. The presence of trypomastigotes in peridomestic ecotopes suggests active parasite circulation in areas of anthropogenic influence, positioning these mammals as key players in local transmission cycles.

### Bat Diversity

Colombia is considered the country with the highest bat diversity in the Neotropics, with a total of 221 species grouped into nine families and 67 genera (nine endemic species), with the families Phyllostomidae and Vespertilionidae being the most numerous ^24^. In the department of Atlántico, bats are the most diverse group of mammals, with 36 species belonging to seven families, with the family Phyllostomidae being the most diverse with 18 species ^25^.

The sampling effort was adequate and uniform across the three localities, allowing for the comparison of alpha diversity. 114 bats from three families and 10 species were captured, representing 27.70% of the species reported in the department of Atlántico ^25^. Phyllostomidae predominated due to the rich vegetation cover in Tubará and Usiacurí, which favors generalist species such as *A. lituratus* and *A. jamaicensis*. In contrast, Piojó has scarce fruit crops, with livestock farming predominating, which explains the presence of *Desmodus rotundus* and the adaptability of *Glossophaga soricina* and *A. lituratus* in altered environments. The families Vespertilionidae and Mormoopidae showed low richness and abundance, likely because mist nets, according to literature, are less effective for capturing insectivorous bats ^15^^,^^26^.

### Frequency of infection by *Trypanosoma* spp. and *Leishmania* spp.

In several American countries, including Brazil, Venezuela, and Colombia, infection by *Trypanosoma* spp. and *Leishmania* spp. has been documented in generalist bats (*A. lituratus*, *P. hastatus*, and *C. perspicillata*), confirming their role as hosts and reservoirs for these parasites ^3^^,^^10^^,^^18^. In Colombia, research on trypanosomatid coinfection is scarce, especially in peridomestic ecotopes of rural areas. The synanthropy of these bats, which use anthropogenic spaces as shelter, increases the probability of contact with humans and the subsequent risk of zoonotic transmission, underlining the importance of strengthening epidemiological surveillance in public health and veterinary medicine ^10^^,^^27^.

Comparing our findings with those of Lourenço *et al*. ^3^ in an endemic area of Brazil, where an infection frequency of 76.00% (111/146) was reported, it is observed that prevalences tend to be higher in high-endemicity areas. Both studies coincide in identifying generalist bats *A. lituratus*, *C. perspicillata*, and *P. hastatus* as hosts in wild and rural environments. Nevertheless, unlike the Brazilian study ^3^, where no parasites were detected by microscopy due to low parasitemia, in the present investigation, trypomastigotes were identified in peripheral blood. The finding of circulating parasites in a territory not traditionally considered endemic suggests the need to update transmission scenarios in the department of Atlántico, recommending the use of xenodiagnosis in future studies to determine the infective potential of these mammals ^3^.

Regarding Colombia, research focuses on the Andean territory and the Orinoquia, with an information bias for the Caribbean region. A study documented by Matiz-González *et al.*^10^ in cave-dwelling bats from the department of Santander reported a trypanosomatid infection frequency of 42.90% (48/112) in bats *C. perspicillata* (19/43; 44.20%), *Natalus tumidirostris* (17/39; 43.60%), and *Mormoops megalophylla* (12/30; 40.00%). Through amplicon sequencing and phylogenetic analysis, the identified trypanosomatids were classified as *Trypanosoma* spp. (33/112; 29.50) and *Leishmania* spp. (8/112; 7.10%), with one individual of the species *C. perspicillata* carrying both trypanosomatid parasites. While the overall infection frequency of the present study is lower, when extrapolating the infection frequencies of the genus *Leishmania*, it was found that the present investigation has a higher infection frequency at 10.00% (11/110) compared to the 7.10% (8/112) reported by Matiz-González *et al*. ^10^; another aspect to highlight is that the present study reports a higher richness of bats infected with trypanosomatids, with five species in contrast to the three in Matiz-González *et al*. ^10^.

The fact that both studies involve the species *C. perspicillata* strengthens the argument of how generalist bat species have a great capacity to adapt to different ecotopes and, therefore, should be a priority in epidemiological surveillance. On the other hand, in the Orinoquia, Zúñiga-González *et al.*^28^ provide the first publication for Colombia of coinfection of *Trypanosoma* spp. and *Leishmania* spp. in bats. Of the total individuals collected, 36.60% (63/172) were positive for *Trypanosoma*, 11.60% (20/172) for *Leishmania*, and 10.40% (18/172) for both parasites; the species with coinfection were *P. hastatus* 66.60% (12/18), *Myotis* spp. 22.20% (4/18), and *C. perspicillata* with 11.10% (2/18). Comparing these findings with the present investigation, it stands out that both share coinfection in *P. hastatus*. Likewise, in both works, *Leishmania* spp. was reported with lower frequency and always associated with a *Trypanosoma* spp. infection, without cases of independent *Leishmania* infection.

Finally, the finding of these parasites in Casanare and Atlántico, departments not traditionally considered endemic for Leishmania, evidences the dynamism of trophic webs and the need to expand research toward historically decentralized territories in Colombia.

At a national level, this study constitutes the first report of coinfection in mainly frugivorous species of the genus *Artibeus*, specifically: *A. jamaicensis* and *A. lituratus*. Coinfection in these species is associated with their feeding preferences, as it has been proven that, although they base their diet on fruits, they can supplement nutritional needs through the consumption of triatomine insects and coexistence with hematophagous arthropods such as sand flies and ticks of the family Ixodidae ^28^^,^^30^. The reason that all cases of *Leishmania* spp. infection occur accompanied by *Trypanosoma* spp. infection suggests a dynamic of cumulative infection. The longevity of bats and their coevolutionary relationship with *Trypanosoma* allow for chronic and persistent infections ^1^^,^^8^^,^^17^^,^^32^. This prolonged presence of the parasite could subtly compromise the host's immune response, facilitating the subsequent establishment of *Leishmania* spp. in habitats where bats are exposed to multiple vectors ^29^^,^^30^.

The present study constitutes the first report of the presence of *Leishmania* spp. and coinfection with *Trypanosoma* spp. in bats from the department of Atlántico, unlike the findings of Marinkelle ^8^ who, in a national study that included municipalities of the Atlantic coast (without specifying), did not report the presence of these protozoa in bats. Subsequently, Benavides-Céspedes *et al.*^9^^)^ reported *Trypanosoma* spp. in bats for the first time for the department of Atlántico, being the pioneer study that allowed the present investigation to cover the presence of trypanosomatids in bats from other municipalities of this department. The detection of trypanosomatids in bats from Atlántico after four decades can be associated with various factors, such as changes in the dynamics of communities and trophic webs, landscape modifications due to anthropogenic activities (population growth, deforestation, urbanization, changes in land use, among others), as well as global warming. All these factors could facilitate the integration of bats into the epidemiological cycles of trypanosomatids, consequently, their role as reservoirs or hosts ^31^.

Another aspect to highlight is the difference in diagnostic methodologies, considering that Marinkelle ^8^ used parasitological diagnosis, while in the present study molecular techniques were employed, which offer higher sensitivity. It is important to highlight that, in the present investigation, blood trypomastigotes were observed through fresh blood examinations; this finding allows us to support the circulation of *Trypanosoma* spp. in bats from the department of Atlántico, which is a high risk factor because the captured bats are surrounding the peridomestic ecotope of rural areas, where the presence of vectors for *Trypanosoma* has already been previously reported ^12^.

Limitations of the study include a relatively small sample size, not expanding bat captures to wild/urban ecotopes, and not performing sampling that would allow comparison of dry and rainy months. The lack of sequencing did not allow establishing which species and which lineages of trypanosomatids are circulating in the study area; nonetheless, the molecular markers used, the report of parasites in blood, and the risk factors associated with the study area are solid arguments to support the presence of trypanosomatids down to the genus level.

In conclusion, this study reports for the first time the presence of *Trypanosoma* spp. and *Leishmania* spp. in bats of the species *A. jamaicensis*, *A. lituratus*, and *P. hastatus* captured in the department of Atlántico, Colombia, expanding knowledge on the frequency of trypanosomatid infection in this region. Likewise, the findings expose new scenarios of *Leishmania* spp. transmission in non-endemic areas of the country, demonstrating the importance of conducting biosanitary research in areas where risk factors converge.
