# Supplementary material for: Detection of Trypanosoma spp. and Leishmania spp. in bats from the department of Atlántico, Colombia
Source: Rev Peru Med Exp Salud Publica. 2026 Mar 27;43(1):6–15. doi: 10.17843/rpmesp.2026.431.15494 (PMC13245985; doi:10.17843/rpmesp.2026.431.15494)
Supplement: Translation

# Translation: Detección de *Trypanosoma* spp. y *Leishmania* spp. en murciélagos del departamento del Atlántico, Colombia

**Authors:** Brendy Alcira Padilla-Obregón, Iván Benavides-Céspedes, Yeisson Cera-Vallejo, Daisy Lozano-Arias, Carlos Mario Meléndez, Roberto Garcia-Alzate

**Affiliations:** 1 Programa de Biología, Facultad de Ciencias Básicas, Universidad del Atlántico, Puerto Colombia, Colombia.; 2 Departamento de Ciencia Animal, Facultad de Ciencias Veterinarias, Universidad de Concepción, Chillán, Chile.; 3 Grupo de Investigación Básica y Clínica en Ciencias de la Salud, Facultad de Ciencias de la Salud, Fundación Universitaria San Martín, Puerto Colombia, Colombia.; 4 Programa de Química, Facultad de Ciencias Básicas, Universidad del Atlántico, Puerto Colombia, Colombia.

**Keywords:** Bats, Host, Natural Infection, Trypanosoma, Leishmania

## Abstract

**Objetivo.:**

Determinar la frecuencia de infección por *Trypanosoma* spp. y *Leishmania* spp. en murciélagos capturados en los municipios de Piojó, Tubará y Usiacurí del departamento del Atlántico, en la región norte del caribe colombiano.

**Materiales y métodos.:**

Entre agosto del 2022 y abril del 2023 se capturaron murciélagos utilizando redes de niebla, estos fueron identificados taxonómicamente y se estableció su sexo, estadio reproductivo y edad relativa. Se obtuvieron muestras de sangre de la vena braquial. La identificación de *Trypanosoma* spp. se realizó mediante diagnóstico parasitológico y molecular, se extrajo ADN y se amplificaron la región del kinetoplasto (kDNA). Para la identificación de *Leishmania* spp. se amplificó la región de la β-tubulina.

**Resultados.:**

De 110 murciélagos analizados, se determinó una frecuencia de infección general del 14,50% (16/110). La presencia de *Trypanosoma* spp. se detectó en el 14,50% (16/110) de los individuos y *Leishmania* spp. en el 10,00% (11/110). Se identificó coinfección en 11 ejemplares, lo que equivale a una frecuencia del 10,00% del total de la muestra. Los murciélagos coinfectados pertenecían a las especies *Artibeus lituratus*, *Artibeus jamaicensis* y *Phyllostomus hastatus*.

**Conclusiones.:**

Este estudio constituye el primer reporte sobre la frecuencia de coinfección por tripanosomátidos en murciélagos del departamento del Atlántico.

## INTRODUCCIÓN

Los murciélagos pertenecen al Orden Chiroptera, considerado el segundo orden más diverso de mamíferos, con 1474 especies reconocidas (https://www.mammaldiversity.org). Esta diversidad se refleja en sus funciones ecológicas (polinización, dispersión de semillas y control de artrópodos) derivadas de sus preferencias alimenticias ^1^. Estas características, junto con el vuelo, longevidad y comportamiento social hacen que los murciélagos sean hospedadores idóneos para virus, bacterias y protozoos, destacando a los hemoflagelados de la familia *Trypanosomatidae*, que incluye géneros de importancia médica y veterinaria (géneros *Trypanosoma* y *Leishmania*) como agentes etiológicos de enfermedades en humanos y otros animales ^(^^2^^-^^4^.

Dentro del género *Trypanosoma*, destaca la especie *Trypanosoma (Schizotrypanum) cruzi* (Kinetoplastea, T*rypanosomatidae*), presentando siete unidades de tipificación discretas (DTUs) TcI - TcVI, incluyendo una DTU específica de murciélagos llamada TcBat, que difieren en su distribución geográfica y ciclos de transmisión ^5^*.* Además, *T. cruzi* puede completar parte de su ciclo de vida en mamíferos de hasta nueve órdenes, incluyendo el Chiroptera ^(^^6^. También es el agente etiológico de la tripanosomiasis americana (TA) o enfermedad de Chagas (ECh), considerada una de las principales causas de morbilidad, discapacidad y mortalidad, principalmente en las Américas, donde es endémica en 21 países, según la Organización Mundial de la Salud (OMS) (2025) ^(^^7^.

El género *Leishmania* incluye 21 especies que afectan a humanos y otros vertebrados, causando leishmaniasis cutánea (LC), leishmaniasis mucocutánea (LMC) y leishmaniasis visceral (LV) ^4^. En Colombia, Marinkelle ^(^^8^ fue el primero en señalar la importancia de los murciélagos como hospedadores de *T. cruzi*, reportando 233 hemocultivos positivos ^8^. Estudios más recientes, como los de Carrasquilla *et a*l. ^(^^4^, Benavides-Céspedes *et al*. ^(^^9^ y Matiz-González *et al*. ^(^^10^ aportan datos actualizados sobre el papel de los murciélagos como hospedadores de tripanosomátidos.

La infección por tripanosomátidos en murciélagos deriva de la interacción trófica y biológica con sus vectores. Mientras T*rypanosoma* spp. se transmite por la ingesta de triatominos infectados o el contacto con sus deyecciones, *Leishmania* spp. se transmite mediante la picadura de flebótomos durante la hematofagia, consolidando una dinámica multidireccional en los ecosistemas ^4^^,^^6^^,^^8^^-^^10^. En Colombia, para la semana epidemiológica 53 del 2025, se reportaron 12 casos de ECh aguda (uno en el Atlántico) y 5721 casos de leishmaniasis ^11^.

En el departamento del Atlántico, se han identificado vectores para *Trypanosoma* spp. como *Panstrongylus geniculatus* y de *Leishmania* como *Lutzomyia longipalpis, Lu. evansi* y *Lu. gomezi* en varios municipios, considerados como factores de riesgo ^12^^,^^13^, también se ha detectado la presencia de *Trypanosoma* spp. en murciélagos de la especie *Molossus molossus* y *Noctilio albiventris*^9^. Dado el creciente contacto humano con animales silvestres, el objetivo de este estudio fue determinar la frecuencia de infección por *Trypanosoma* spp. y *Leishmania* spp. en murciélagos de ecótopos peridomiciliarios en el departamento del Atlántico.

**MENSAJE CLAVE**

Motivación para realizar el estudio. En el departamento del Atlántico se documentó el primer reporte de tripanosomátidos en murciélagos, por ello, resulta necesario ampliar este conocimiento en otros municipios de la región.

Principales hallazgos. Este estudio constituye el primer reporte de la frecuencia de coinfección por tripanosomátidos y *Leishmania* spp. en murciélagos de los géneros *Artibeus* y *Phyllostomus* en el departamento del Atlántico.

Implicancias en salud pública. La identificación de murciélagos infectados con *Trypanosoma* spp. y *Leishmania* spp. en municipios con presencia de vectores biológicos para estos parásitos representa un potencial riesgo para la salud pública, debido a su posible papel en la transmisión zoonótica.

## MATERIALES Y MÉTODOS

### Tipo y área de estudio

El presente estudio es de tipo observacional, descriptivo y de corte transversal; el muestreo se realizó entre agosto de 2022 y abril de 2023 en los ecótopos peridomiciliarios de tres municipios del departamento del Atlántico (10° 39’ 00’’ N, 74° 58’ 00’’ O), ubicado en el norte de Colombia, a 123 m s.n.m. y temperatura promedio de 27,1 °C y una precipitación de 1396 mm ^14^. Los municipios fueron Usiacurí, Piojó y Tubará; estos fueron escogidos por presentar factores de riesgo como la presencia de mamíferos hospederos (datos no publicados) y triatominos positivos para *T. cruzi*^12^.

El área de estudio se encuentra bajo la clasificación climática de Köppen-Geiger como ecuatorial de sabana con invierno seco (Aw), bajo este marco, el municipio de Piojó (10º 45’ 01” N, 75º 06’ 27” O) presenta una temperatura media anual de 26 °C y una altitud de 240 m s.n.m., con una precipitación promedio anual de 1200 mm y un régimen bimodal compuesto por dos periodos lluviosos (mayo-junio y agosto-noviembre) y dos secos (diciembre-abril y junio-julio), lo que lo convierte en uno de los municipios más lluviosos y altos del Atlántico. Usiacurí (10° 44’ 46’’ N, 74° 59’ 02’’O), situado a una altitud de 95 m s.n.m., registra una temperatura media anual entre 25 °C y 30 °C y una precipitación de 980 mm anuales, con picos de lluvia en los periodos de mayo-junio y septiembre-octubre. 

Finalmente, Tubará (10° 52’ 27’’ N, 74° 58’ 43’’ O), ubicado a 192 m s.n.m., registra una temperatura anual promedio entre 26 °C y 29 °C y una precipitación que oscila entre 379 mm y 1025 mm anuales; su temporada de lluvias inicia en abril y culmina a principios de diciembre, siendo septiembre, octubre y noviembre los meses de mayor pluviosidad (figura 1).

**Figura 1:** Distribución geográfica de los tres municipios donde se capturaron especies de murciélagos en el Departamento del Atlántico.

Fuente: elaboración propia.

### Captura, identificación taxonómica, toma de sangre y marcaje de murciélagos

Se realizaron dos visitas de dos noches consecutivas por municipio, sumando un acumulado total de 12 noches. Para la captura de los murciélagos se usaron dos redes de niebla de 12 x 2,5 m, con ojo de malla de 3 × 3 cm (BioWed®), que se extendieron entre las 18:00 y las 23:00 horas en dos puntos específicos (seleccionados mediante visitas piloto) del ecótopo peridomiciliario para cada municipio, con una distancia entre redes de 25 metros; cada punto fue geolocalizado con un GPS (Garmin Etrex 20). Estas se monitorearon cada 15 minutos para minimizar el posible estrés en los animales ^15^. Los individuos extraídos de las redes fueron depositados individualmente en bolsas de algodón de 20 × 20 × 20 cm para su posterior identificación, utilizando la clave taxonómica de Díaz *et al.*^16^. Se determinó el sexo, la edad relativa y el estado reproductivo según las indicaciones de Kunz y Wemmer ^17^.

Para la detección, se colectaron 100 μL de sangre de la vena braquial, divididos en dos alícuotas de 50 μL: una en EDTA a 4 °C para análisis molecular y otra para examen parasitológico *in situ*. Este último consistió en la búsqueda de hemoflagelados compatibles con *Trypanosoma* spp. ^18^ mediante gota gruesa (Giemsa) y microscopía de campo claro (40X). Tras el muestreo, se realizaron microperforaciones en el patagio derecho para identificar individuos y evitar recapturas antes de su liberación.

Una vez verificado que el murciélago estaba en condiciones adecuadas para volar, se liberó cerca del lugar de captura. Se excluyó a las hembras gestantes y lactantes de los análisis parasitológicos y moleculares. Los datos de cada individuo se registraron en una planilla propia. Esta investigación fue aprobada por el Comité de Ética de la Universidad del Atlántico bajo el código 02-III-2021, conforme a la resolución 1214 de la Autoridad Nacional de Licencias Ambientales (ANLA) para investigaciones científicas sin fines comerciales en Puerto Colombia.

### Detección parasitológica y molecular

La extracción de ADN de las muestras de sangre almacenadas en tubos con EDTA se realizó con el kit de purificación HMW Wizard® (Promega Corporation: Madison, WI, USA), siguiendo las instrucciones del fabricante a partir de 50 uL de sangre y eludido en un volumen final de 20 uL. La concentración del ADN genómico fue determinada empleando el espectrofotómetro EPOCH 2NS (BioTeck Instruments, Inc: USA). La detección molecular de *Trypanosoma* spp. se realizó mediante la amplificación por PCR de la región conservada del minicirculo del kinetoplasto, utilizando los cebadores 121 (AAA-TAA-TGT-ACG-GG(T/G)-GAG-ATG-CAT-GA) y 122 (GGT-TCG-ATT-GGG-GTT-GGT-GTA-ATA-TA), que amplifican una banda de 330 pb ^19^. Para la detección molecular del género *Leishmania*, se realizó una PCR dirigida a la región de la β-tubulina usando los cebadores TUB 1 (ATG-CGT-GAG-ATC-GTT-TCC) y TUB 6 (GGC-GGC-CTG-CAT-CAT); las muestras se consideraron positivas cuando se amplificaba una banda de 900 pb ^20^. En cada reacción de PCR se utilizó agua ultrapura como control sin molde (NTC - No Template Control). Como controles positivos se emplearon ADN genómico de una cepa de referencia local de *T. cruzi* y la cepa de referencia de la OMS *L.* (Viannia) *panamensis* (MHOM/PA/71/LS94), para la detección de *Trypanosoma spp*. y *Leishmania* spp., respectivamente. Previamente se realizaron controles de cada marcador molecular para garantizar que no existieran reacciones cruzadas.

Las reacciones de PCR se estandarizaron utilizando la mezcla PCR Master Mix (Promega®). Para la detección de *Trypanosoma* spp., se empleó un volumen final de 15 μL (5 μL de ADN molde), añadiendo con una concentración final de 0,016 U/μL de Taq DNA polimerasa, 0,66 μM de cada cebador y una suplementación de 1,6 mM de MgCl₂ adicional a los 1,5 mM presentes en la mezcla comercial. Para el género *Leishmania*, el volumen final fue de 20 μL (5 μL de ADN molde), ajustando las concentraciones a 0,015 U/μL de Taq DNA polimerasa, 1,0 μM de cada cebador y una suplementación de 1,25 mM de MgCl₂. En ambos casos, se utilizó agua libre de nucleasas (Promega®) para completar el volumen final.

Cada PCR se realizó en un termociclador TC-9639 (Benchmark SCIENTIFIC, Sayreville, NJ, USA) bajo las siguientes condiciones: Para Trypanosoma *spp.* desnaturalización inicial a 94 ºC por 10 minutos, 35 ciclos de desnaturalización a 94 ºC/1 minuto, hibridación a 63 ºC/1 minuto, extensión 72 ºC/1 minuto y extensión final a 72 ºC por 10 minutos ^19^. Para el género *Leishmania*: desnaturalización inicial a 94 °C por 5 minutos, 40 ciclos de desnaturalización a 94 °C/30 segundos, hibridación a 62 °C/ 30 segundos, extensión a 72 °C/30 segundos y una extensión final a 72 °C/10 minutos ^20^.

Los productos obtenidos se evidenciaron por electroforesis horizontal (80V/60 minutos) en gel de agarosa al 2,0%, teñidos con bromuro de etidio diluido en buffer TAE 1X durante 20 minutos para su visualización y fotodocumentación en el sistema iBright™. FL1500 (Thermo Fisher Scientific Inc: MA, USA), se utilizó un marcador molecular de 100 a 1.000 pb (Biotech) como referencia.

### Análisis de datos

El esfuerzo de muestreo total/por noche y el éxito de captura se calculó según las recomendaciones generales para muestreos con redes de niebla, la frecuencia de infección se calculó en porcentajes utilizando la fórmula estándar, mientras que la diversidad alfa se estimó mediante la serie de órdenes de abundancia y diversidad de Hill (con q=0 se obtiene la riqueza directamente, para q=1 especies abundantes, y para q=2 especies muy abundantes o dominantes) ^21^. Se realizaron curvas de rarefacción para cada uno de los órdenes, que se interpolaron y extrapolaron al número mínimo y máximo de individuos recolectados en cada localidad, lo que permite comparar sitios con distintos números de individuos, y cuyo sombreado indica los intervalos de confianza del 95% ^22^. Todos los cálculos de diversidad alfa se realizaron en Excel y el paquete iNEXT del programa estadístico R ^23^.

La prueba exacta de Fisher (bilateral) se utilizó para evaluar si las frecuencias de infección por tripanosomátidos totales entre las dos zonas (Usiacurí y Tubará) eran significativamente diferentes. Los análisis se realizaron en IBM SPSS Statistics versión 25 y se empleó un nivel de confianza del 95% (α =0,05). Se reportaron las proporciones de infección por área, la diferencia de proporciones y su intervalo de confianza al 95%.

### Aspectos éticos

Se siguieron todas las directrices internacionales, nacionales y/o institucionales aplicables para el cuidado y uso de animales. El estudio fue aprobado por el Comité de Ética de la Universidad del Atlántico, Puerto Colombia, Colombia, con el código 02-IV-2022.

## RESULTADOS

### Esfuerzo de muestreo total, esfuerzo de muestreo por noche, éxito de captura y diversidad de especies de murciélagos

El esfuerzo de muestreo total del estudio fue de 3600 h/red. En cuanto al esfuerzo de muestreo por noche, este fue de 150 h/red, siendo homogéneo para cada zona, mientras que el éxito de captura para Usiacurí fue de 0,34 ind/h-red, en Tubará, fue de 0,26 ind/h-red y en el municipio de Piojó fue de 0,15 ind/h-red.

Se capturaron un total de 114 murciélagos (no se registraron recapturas) (N=114) representados en tres familias y 10 especies. La familia *Phyllostomidae* fue la más abundante, representando el 96,50% (110/114) del total de capturas, las familias *Vespertilionidae* (2,62% - 3/114) y *Mormoopidae* (0,88% - 1/114) estuvieron poco representadas. Las especies más abundantes *fueron A. lituratus* (41,23% - 47/114) y *A. jamaicensis* (35,09% - 40/114), aportando el género *Artibeus* el 76,32% del total de individuos, lo distribución de los murciélagos por municipio se puede observar en la tabla 1.

**Tabla 1:** Gremio trófico, estado reproductivo y edad de las especies recolectadas en las áreas de estudio.

Área	Familia	Especies	Gremio trófico	Estado reproductivo									Edad M				Edad H				Total
				Msa	Mnas	Total	Has	Hnas	Hg	Hl	Hcc	Total	A	SA	J	Total	A	SA	J	Total	
Piojó	*Phyllostomidae*	*Artibeus lituratus*	P.F	7	1	8	2	0	1	0	0	2	7	0	1	8	1	1	0	2	10
		*Glossophaga soricina*	Nc	5	0	5	2	2	0	1	0	4	5	0	0	5	1	1	2	4	9
		*Desmodus rotundus*	Hm	2	0	2	0	0	0	0	0	0	2	0	0	2	0	0	0	0	2
	*Vespertilionidae*	*Myotis riparius*	In	0	0	0	2	0	0	0	0	2	0	0	0	0	2	0	0	2	2
	Total			14	1	15	6	2	1	1	0	8	14	0	1	15	4	2	2	8	23
Tubará	*Phyllostomidae*	*Artibeus lituratus*	P.F	6	0	6	10	2	0	0	0	12	5	1	0	6	8	2	2	12	18
		*Artibeus jamaicensis*	P.F	5	3	8	3	3	0	0	0	6	5	0	3	8	3	0	3	6	14
		*Carollia perspicillata*	Fr	1	0	1	0	4	0	0	0	4	1	0	0	1	0	0	4	4	5
		*Glossophaga soricina*	Nc	0	0	0	0	1	0	0	0	1	0	0	0	0	0	0	1	1	1
	*Vespertilionidae*	*Myotis riparius*	In	0	0	0	0	1	0	0	0	1	0	0	0	0	0	0	1	1	1
	*Mormoopidae*	*Pteronotus gymnonotus*	In	0	1	1	0	0	0	0	0	0	0	0	1	1	0	0	0	0	1
	Total			12	4	16	13	11	0	0	0	24	11	1	4	16	11	2	11	24	40
Usiacurí	*Phyllostomidae*	*Artibeus jamaicensis*	P.F	9	4	13	7	6	0	0	0	13	7	2	4	13	5	2	6	13	26
		*Artibeus lituratus*	P.F	9	2	11	4	4	0	1	0	8	8	1	2	11	4	0	4	8	19
		*Phyllostomus hastatus*	Om	1	0	1	0	0	0	0	0	0	1	0	0	1	0	0	0	0	1
		*Phyllostomus discolor*	In	1	0	1	0	0	0	0	0	0	1	0	0	1	0	0	0	0	1
		*Glossophaga soricina*	Nc	0	0	0	2	1	1	0	0	3	0	0	0	0	1	1	1	3	3
		*Uroderma convexum*	Fr	1	0	1	0	0	0	0	0	0	1	0	0	1	0	0	0	0	1
	Total			21	6	27	13	11	1	1	0	24	18	3	6	27	10	3	11	24	51

M: macho, H: hembra, Msa: macho sexualmente activo, Mnas: macho no activo sexualmente, Hsa: hembra sexualmente activa, Hnas: hembra no activa sexualmente, Hg: hembra gestante, Hl: hembra lactante, Hcc: hembra con crías, A: adulto, SA: subadulto, J: juvenil. P.F: predominantemente frugívoro, Nc: nectarívoro, Hm: hematófago, In: insectívoro, Fr: frugívoro, Om: omnívoro.

La mayoría de los murciélagos capturados fueron adultos (59,60%, 68/114), seguidos de juveniles (30,70%, 35/114) y subadultos (9,60%, 11/114). En cuanto al sexo, el 50,80% (58/114) eran machos y el 49,10% (56/114) hembras. No se encontraron hembras con crías, pero se capturaron dos hembras lactantes y dos gestantes. Los detalles por área se presentan en la tabla 1.

Las curvas de rarefacción mostraron que la riqueza (q=0) para Usiacurí y Tubará fue homogénea, con seis especies, y con un mayor esfuerzo de muestreo podrían registrarse más. Piojó presentó la menor riqueza, con cuatro especies, y su curva se estabilizó, sugiriendo que no se agregarían más especies con un muestreo mayor. Las diferencias en riqueza entre los municipios no fueron estadísticamente significativas (α = 0,05). En cuanto a la abundancia (q=1), Tubará y Piojó mostraron similitudes, sin diferencias significativas; la cantidad de especies abundantes fue adecuada en todos los municipios. Para el número efectivo de especies dominantes (q=2), Piojó y Tubará también mostraron similitudes, con mayor equidad en comparación con Usiacurí, aunque sin diferencias significativas (figura 2).

**Figura 2:** Curvas de rarefacción para cada orden de diversidad en los municipios de Piojó, Tubará y Usiacurí. La línea sólida representa la región interpolada u observada, la línea punteada representa la región extrapolada y el sombreado alrededor de cada curva corresponde al intervalo de confianza del 95% (IC).

### Frecuencia de infección

De los 114 murciélagos capturados, se analizaron 110 (96,50%), debido al criterio de exclusión de hembras gestantes y lactantes mencionado anteriormente. Así, empleando el método parasitológico, la frecuencia de infección obtenida fue del 7,30% (8/110), distribuida entre los municipios de Usiacurí (62,50% - 5/8) y Tubará (37,50% - 3/8); los individuos con presencia de tripomastigotes sanguícolas pertenecían a las especies *P. hastatus*, *A. jamaicensis* y *Pteronotus gymnonotus*, los detalles se especifican en la tabla 2.

**Tabla 2:** Frecuencia de infección en general y por especies de murciélagos obtenidas en cada una de las áreas de estudio.

Área	% Parasitológico (n/N) IC 95%	% Molecular (n/N) IC 95%	Especies	*Trypanosoma sanguícola*	*Trypanosoma* (Mol)	*Leishmania*	Coinfección
Usiacurí	10,20 (5/49) 4,43‒21,97	22,45 (11/49) 13,01‒35,93	*A. jamaicensis*	15,38 (4/26) 6,14‒33,53	23,08 (6/26) 10,91‒42,26	23,08 (6/26) 10,91‒42,26	23,08 (6/26) 10,91‒42,26
			*A. lituratus*	0,00 (0/18) 0,00‒17,59	22,22 (4/18) 8,97‒45,18	22,22 (4/18) 8,97‒45,18	22,22 (4/18) 8,97‒45,18
			*P. hastatus*	100,00 (1/1) 20,65‒100,00	100,00 (1/1) 20,65‒100,00	100,00 (1/1) 20,65‒100,00	100,00 (1/1) 20,65‒100,00
Tubará	7,50 (3/40) 2,58‒19,85	12,50 (5/40) 5,45‒25,95	*A. jamaicensis*	14,29 (2/14) 4,00‒40,03	7,14 (1/14) 1,27‒31,48	0,00 (0/14) 0,00‒21,50	0,00 (0/14) 0,00‒21,50
			*A. lituratus*	0,00 (0/18) 0,00‒17,59	11,11 (2/18) 3,11‒32,80	0,00 (0/18) 0,00‒17,59	0,00 (0/18) 0,00‒17,59
			*C. perspicillata*	0,00 (0/5) 0,00‒45,93	20,00 (1/5) 3,62‒62,45	0,00 (0/5) 0,00‒45,93	0,00 (0/5) 0,00‒45,93
			*Pt. gymnonotus*	100,00 (1/1) 20,65‒100,00	100,00 (1/1) 20,65‒100,00	0,00 (0/1) 0,00‒79,35	0,00 (0/1) 0,00‒79,35
Piojó	0,00 (0/21) 0,00‒15,33	0,00 (0/21) 0,00‒15,33		0,00 (0/21) 0,00‒15,33	0,00 (0/21) 0,00‒15,33	0,00 (0/21) 0,00‒15,33	0,00 (0/21) 0,00‒15,33
Total	7,27 (8/110) 3,73‒13,71	14,55 (16/110) 9,13‒22,33		7,27 (8/110) 3,73‒13,71	14,55 (16/110) 9,13‒22,33	10,00 (11/110) 5,66‒17,04	10,00 (11/110) 5,66‒17,04

IC 95%: Intervalo de Confianza del 95% calculado mediante el método de Wilson.

En cuanto a la detección molecular, la frecuencia de infección por tripanosomátidos fue del 14,50% (16/110). La totalidad de estos individuos positivos (100%) presentó infección por *Trypanosoma* spp. (figura 3A -B). Asimismo, en 11 de estos 16 ejemplares se detectó la presencia de *Leishmania* spp. (muestras 1-11, figura 4), lo que representa una prevalencia del 10,00% (11/110) para este género. Cabe destacar que todos los casos positivos para *Leishmania* spp. correspondieron a eventos de coinfección con *Trypanosoma* spp.

**Figura 3:** (A) Gel de agarosa al 2% en 1X TAE teñido con bromuro de etidio. Amplificación por PCR de kDNA de 121-122. Muestras positivas: (1,2,5,7-13). (B) Gel de agarosa al 2% en 1X TAE teñido con bromuro de etidio. Amplificación por PCR de kDNA de 121-122. Muestras positivas: (14-19). MPM: marcador de peso molecular. NTP: control negativo (agua ultrapura). CP: control positivo (ADN genómico de una cepa de referencia local de *Trypanosoma cruzi*-DVP50).

**Figura 4:** Gel de agarosa al 2% en 1X TAE teñido con bromuro de etidio. Amplificación por PCR TUB1/TUB6. Muestras positivas: 1-11. NTP: control negativo (agua ultrapura). CP: control positivo *(Leishmania (Viannia) panamensis*- MHOM/PA/71/LS94).

### Comparación de las frecuencias de infección entre las áreas de estudio

Con respecto a la frecuencia de infección y coinfección por zonas, el municipio de Usiacurí presentó la mayor frecuencia de infección global (tripanosomátidos) y coinfección con un 22,45% (11/49), seguido del municipio de Tubará, donde la frecuencia de infección global fue del 12,50% (5/40), sin casos de coinfección. Finalmente, el municipio de Piojó no registró individuos positivos. Las especies con mayor frecuencia de infección fueron *P. hastatus* y *Pt. Gymnonotus*; la especie *P. hastatus* también presentó la mayor frecuencia de coinfección, todos los detalles se agrupan en la tabla 2.

Al comparar la frecuencia de infección por tripanosomátidos entre murciélagos de Usiacurí (22,40%; 11/49) y Tubará (12,50%; 5/40) mediante la prueba exacta de Fisher (bilateral), no se detectó una diferencia estadísticamente significativa (*p*=0,332). La proporción fue mayor en Usiacurí; la diferencia de proporciones fue 0,0995 (IC 95%: −0,0610 a 0,2600).

## DISCUSIÓN

En el presente estudio se determinó una frecuencia global de infección por tripanosomátidos del 14,55% mediante técnicas moleculares, cifra que duplicó la detección por microscopía directa (7,27%). Se destaca el hallazgo de una coinfección del 10,00% entre *Trypanosoma* spp. y *Leishmania* spp. en murciélagos generalistas (*A. lituratus*, *A. jamaicensis* y *P. hastatus*), lo que constituye el primer registro de coinfección natural y la primera detección de *Leishmania* spp. en murciélagos para el departamento del Atlántico. La presencia de tripomastigotes en ecótopos peridomiciliarios sugiere una circulación parasitaria activa en áreas de influencia antrópica, posicionando a estos mamíferos como actores clave en los ciclos de transmisión local.

### Diversidad de murciélagos

Colombia es considerado el país de mayor diversidad de murciélagos en el neotrópico, con un total de 221 especies, agrupadas en nueve familias y 67 géneros (nueve especies endémicas), siendo las familias *Phyllostomidae* y *Vespertilionidae* las más numerosas ^24^. En el departamento del Atlántico, los murciélagos son el grupo más diverso de mamíferos, con 36 especies pertenecientes a siete familias, siendo la familia *Phyllostomidae* la más diversa con 18 especies ^25^.

El esfuerzo de muestreo fue adecuado y uniforme en las tres localidades, permitiendo comparar la diversidad alfa. Se capturaron 114 murciélagos de tres familias y 10 especies, lo que representa el 27,70% de las especies reportadas en el departamento del Atlántico ^25^. *Phyllostomidae* predominó debido a la rica cobertura vegetal en Tubará y Usiacurí, que favorece a especies generalistas como *A. lituratus* y *A. jamaicensis*. En contraste, Piojó tiene escasos cultivos frutales, predominando la ganadería, lo que explica la presencia de *Desmodus rotundus* y la adaptabilidad de *Glossophaga soricina* y *A. lituratus* en entornos alterados. Las familias *Vespertilionidae* y *Mormoopidae* mostraron baja riqueza y abundancia, probablemente debido a que las redes de niebla, según la literatura, son poco efectivas para capturar murciélagos insectívoros ^15^^,^^26^.

### Frecuencia de infección por *Trypanosoma* spp. y *Leishmania* spp.

En varios países de América, incluidos Brasil, Venezuela y Colombia, se ha documentado la infección por *Trypanosoma* spp. y *Leishmania* spp. en murciélagos generalistas (*A. lituratus, P. hastatus* y *C. perspicillata*), confirmando su rol como hospedadores y reservorios de estos parásitos ^3^^,^^10^^,^^18^. En Colombia, las investigaciones sobre coinfección por tripanosomátidos son escasas, especialmente en ecótopos peridomiciliarios de áreas rurales. La sinantropía de estos murciélagos, que utilizan espacios antrópicos como refugio, incrementa la probabilidad de contacto con humanos y el consecuente riesgo de transmisión zoonótica, lo que subraya la importancia de fortalecer la vigilancia epidemiológica en salud pública y veterinaria ^10^^,^^27^.

Al comparar nuestros hallazgos con los de Lourenço *et al*. ^3^ en una zona endémica de Brasil, donde se reportó una frecuencia de infección del 76,00% (111/146), se observa que las prevalencias tienden a ser superiores en áreas de alta endemicidad. Ambos estudios coinciden en identificar a los murciélagos generalistas *A. lituratus*, *C. perspicillata* y *P. hastatus* como hospedadores en ambientes silvestres y rurales. No obstante, a diferencia del estudio en Brasil ^3^, donde no se detectaron parásitos por microscopía debido a una baja parasitemia, en la presente investigación se identificaron tripomastigotes en sangre periférica. El hallazgo de parásitos circulantes en un territorio no considerado tradicionalmente endémico sugiere la necesidad de actualizar los escenarios de transmisión en el departamento del Atlántico, recomendándose el uso de xenodiagnóstico en futuros estudios para determinar el potencial infectante de estos mamíferos ^3^.

Con respecto a Colombia, las investigaciones se centran en territorio Andino y la Orinoquia, existiendo un sesgo de información para la región caribe. Un estudio documentado por Matiz-González *et al*. ^(^^10^ en murciélagos cavernícolas del departamento de Santander, reportó una frecuencia de infección del 42,90% (48/112) por tripanosomátidos, en los murciélagos *C. perspicillata* (19/43; 44,20%), *Natalus tumidirostris* (17/39; 43,60%) y *Mormoops megalophylla* (12/30; 40,00%). Mediante la secuenciación de amplicones y el análisis filogenético, los tripanosomátidos identificados se clasificaron como *Trypanosoma* spp. (33/112; 29,50) y *Leishmania* spp. (8/112; 7,10%) siendo un individuo de la especie *C. perspicillata* portador de ambos parásitos de tripanosomátidos. Si bien, la frecuencia de infección general del presente estudio es menor, cuando se extrapolan las frecuencias de infección del género *Leishmania*, se obtuvo que la presente investigación tiene mayor frecuencia de infección con un 10,00% (11/110) comparada con el 7,10% (8/112) reportado por Matiz-González *et al*.^10^; otro aspecto a resaltar es que el presente estudio reporta mayor riqueza de murciélagos infectados con tripanosomátidos, con cinco especies en contraste con las tres de Matiz-González *et al*.^10^.

El hecho de que ambos estudios involucran a la especie *C. perspicillata* fortalece el argumento de como las especies de murciélagos generalistas tienen gran capacidad de adaptación a diferentes ecótopos, y por ende deben ser prioridad en la vigilancia epidemiológica. Por otra parte, en la Orinoquia, Zúñiga-González *et al.* ^(^^28^*,* realiza la primera publicación para Colombia de coinfección de *Trypanosoma* spp. y *Leishmania* spp. en murciélagos. Del total de los individuos recolectados, 36,60% (63/172) resultaron positivos para T*rypanosoma*, 11,60% (20/172) para *Leishmania*, y 10,40% (18/172) para ambos parásitos; las especies con coinfección fueron *P. hastatus* 66.60% (12/18), *Myotis* spp. 22,20% (4/18), y *C. perspicillata* con 11,10% (2/18). Al comparar estos hallazgos con la presente investigación, destaca que ambas comparten la coinfección en *P. hastatus*. Asimismo, en ambos trabajos*, Leishmania* spp. se reportó con menor frecuencia y siempre asociada a una infección por *Trypanosoma* spp., sin casos de infección independiente por *Leishmania*.

Finalmente, el hallazgo de estos parásitos en Casanare y Atlántico, departamentos no considerados tradicionalmente endémicos para leishmania, evidencia el dinamismo de las tramas tróficas y la necesidad de ampliar la investigación hacia territorios históricamente descentralizados en Colombia.

A nivel nacional, este estudio constituye el primer reporte de coinfección en especies principalmente frugívoras del género *Artibeus*, específicamente: *A. jamaicensis* y *A. lituratus*. La coinfección en estas especies se asocia a sus preferencias alimenticias, ya que se ha comprobado que, aunque basan su dieta en frutas, pueden suplir necesidades nutricionales mediante el consumo de insectos triatominos, y a la coexistencia con artrópodos hematófagos como flebótomos y garrapatas de la familia *Ixodidae*^28^^,^^30^. La razón de que todos los casos de infección por *Leishmania* spp. se den acompañados de infección por *Trypanosoma* spp. sugiere una dinámica de infección acumulativa. La longevidad de los murciélagos y su relación coevolutiva con *Trypanosoma* permiten infecciones crónicas y persistentes ^1^^,^^8^^,^^17^^,^^32^. Esta presencia prolongada del parásito podría comprometer sutilmente la respuesta inmune del hospedador, facilitando el establecimiento posterior de *Leishmania* spp. en hábitats donde los murciélagos están expuestos a múltiples vectores ^29^^,^^30^.

El presente estudio constituye el primer reporte de la presencia de *Leishmania* spp. y coinfección con *Trypanosoma s*pp. en murciélagos del departamento del Atlántico, a diferencia de los hallazgos de Marinkelle ^8^^)^ quien, en un estudio nacional, que incluyó municipios de la costa Atlántica (sin especificar), no reportó la presencia de estos protozoos en murciélagos. Posteriormente, Benavides-Céspedes *et al.* ^(^^9^ reportaron por primera vez *Trypanosoma* spp. en murciélagos para el departamento del Atlántico, siendo este el estudio pionero para que en la presente investigación se pudiera abarcar la presencia de tripanosomátidos en murciélagos de otros municipios de este departamento. La detección de tripanosomátidos en murciélagos del Atlántico después de cuatro décadas puede asociarse a diversos factores, como cambios en la dinámica de comunidades y redes tróficas, modificaciones del paisaje por actividades antropogénicas (crecimiento poblacional, deforestación, urbanización, cambios en el uso del suelo, entre otros), así como el calentamiento global. Todos estos factores podrían facilitar la integración de los murciélagos en los ciclos epidemiológicos de tripanosomátidos, en consecuencia, su papel como reservorios u hospederos ^31^.

Otro aspecto para destacar es la diferencia en las metodologías diagnósticas, considerando que Marinkelle ^8^^)^ utilizó diagnóstico parasitológico, mientras que en el presente estudio se emplearon técnicas moleculares, las cuales ofrecen mayor sensibilidad. Es importante resaltar que, en la presente investigación, se observaron tripomastigotes sanguícolas a través de exámenes de sangre fresca, este hallazgo nos permite respaldar la circulación de *Trypanosoma* spp. en murciélagos del departamento del Atlántico, lo cual es un factor de riesgo alto, porque los murciélagos capturados, están circundando el ecótopo peridomiciliario de áreas rurales, en donde ya se ha reportado previamente la presencia de vectores para *Trypanosoma*^12^.

Dentro de las limitaciones del estudio se menciona un tamaño de muestra relativamente pequeño, no expandir las capturas de murciélagos a ecótopos silvestres/urbanos y no realizar muestreos que permitieran la comparación de meses secos y lluviosos. La falta de secuenciación no permitió establecer que especies y que linajes de tripanosomátidos están circundando en el área de estudio, no obstante, los marcadores moleculares usados, el reporte de parásitos en sangre y los factores de riesgo asociados al área de estudio son argumentos sólidos para sustentar la presencia de tripanosomátidos hasta el nivel de género.

En conclusión, este estudio reporta por primera vez la presencia de*Trypanosoma*spp. y*Leishmania*spp. en murciélagos de las especies*A. jamaicensis*,*A. lituratus*y*P. hastatus*capturados en el departamento del Atlántico, Colombia, expandiendo el conocimiento sobre la frecuencia de infección por tripanosomátidos en esta región. Asimismo, los hallazgos exponen nuevos escenarios de transmisión de*Leishmania*spp. en zonas no endémicas del país, demostrando la importancia de realizar investigaciones biosanitarias en áreas donde convergen factores de riesgo.
